# The effectiveness of a novel treatment of TIM‐3(−) NK cells infusion in murine models of immune‐mediated bone marrow failure

**DOI:** 10.1002/jcla.24944

**Published:** 2023-08-04

**Authors:** Shaoxue Ding, Tian Zhang, Zixuan Liu, Yi Cui, Chunyan Liu, Rong Fu

**Affiliations:** ^1^ Department of Hematology Tianjin Medical University General Hospital Tianjin China

**Keywords:** aplastic anemia, BMF model, natural killer cells, TIM3

## Abstract

**Background:**

T‐cell immunoglobulin and mucin‐containing domain (TIM)‐3 exerts its inhibitory effect on NK cells and participates in the immune pathogenesis of SAA. In this study, we aimed to explore a novel treatment method of TIM‐3(+) NK or TIM‐3(−) NK cell infusion in combination with immunosuppressive therapy for bone marrow failure (BMF)/aplastic anemia (AA) mice.

**Methods:**

BMF/AA mouse model was constructed. The TIM‐3 expression and functional molecules on TIM‐3(+) and TIM‐3(−) NK cells of the BMF group, total body irradiation (TBI) group, and normal control (NC) group mice were detected by flow cytometry. After treatment, the general condition, whole blood cell and bone marrow cell (BMC) count, and immune condition of mice from each group were compared.

**Results:**

TIM‐3 expression in the peripheral blood NK cells of BMF mice was significantly lower than that of the TBI and NC group mice. TIM‐3(−) NK cells expressed more NKG2D receptors than TIM‐3(+) NK cells. The levels of P‐Akt and PI3K in TIM‐3(−) NK cells were higher than those in TIM‐3(+) NK cells. On the 17th day after BMF induction, the weight, peripheral whole blood cell count, and BMC count of BMF mice decreased significantly compared with that of the NC group mice. The therapeutic effect in the TIM‐3(−) NK cell treatment group was better than that in the TIM‐3(+) NK cell treatment and CsA treatment groups. Concurrently, the ratio of CD4^+^T and CD8^+^T cells of BMF mice was significantly lower than that of the NC group mice. The therapeutic effect in CsA + TIM‐3(−) NK group was more significant than that of the CsA treatment and the CsA + TIM‐3(+) NK groups.

**Conclusions:**

In this study, we found that the general condition, peripheral whole blood cell and BMC count, and immune status of BMF mice improved significantly after CsA + TIM‐3(−) NK cell treatment. These results may provide further insights into the immune pathogenesis of SAA and novel therapeutic ideas for improving SAA treatment.

## Graphical abstract

BMF/AA mouse model was constructed. After BMF induction, BMF mice were divided into the following seven groups based on the given treatment: AA group, CsA treatment group, TIM‐3(+) NK cells treatment group, TIM‐3(−) NK cells treatment group, CsA combined with TIM‐3(−) NK cells treatment (CsA + TIM3–NK) group, TIM‐3 blocker treatment (TIM‐3 blocker) group, and CsA combined with TIM‐3 blocker treatment (CsA + TIM‐3 blocker) group. After treatment, the general condition, whole blood cell and BMC count and immune condition of mice in each group were compared. The therapeutic effect in CsA + TIM‐3(−) NK group was more significant than other groups.

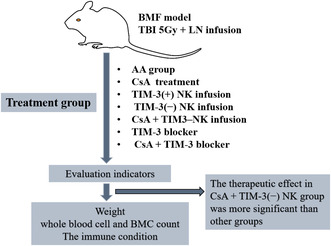

## 1 INTRODUCTION

Aplastic anemia (AA) is a bone marrow failure (BMF) disorder resulting in bone marrow hypocellularity and peripheral pancytopenia. Abnormal immune system activation plays a key role in AA pathogenesis, and immunosuppressive therapy (IST) using antithymocyte globulin (ATG) and cyclosporine A (CsA) can effectively treat the disease.^1^, ^2^ The currently recognized AA pathogenesis includes a complex immune network where a proportion of natural killer (NK) cells that suppress the negative regulatory factors of immune response is significantly decreased.^3^, ^4^, ^5^, ^6^ Even with the application of IST and the new drug eltrombopag (ELT), 30% of patients still have poor treatment effects, 10% of effective patients have a recurrence possibility, and 16% of patients have treatment‐related death because of drug‐related side effects.^7^, ^8^ Therefore, further exploration of the role of NK cells and their subgroups in SAA pathogenesis can be helpful in discovering novel therapeutic targets and improve therapeutic efficacy.^9^, ^10^, ^11^ In our previous study, we reported a decrease in NK cell numbers in patients with SAA, which played a protective role in the onset of SAA.^9^, ^10^, ^11^ We also found low T‐cell immunoglobulin and mucin‐containing domain (TIM)‐3 expression in NK cells in newly‐diagnosed patients with SAA compared with healthy controls and a negative correlation of TIM‐3 expression with the severity of pancytopenia, which further laid the theoretical foundation for improving the efficacy of NK cell reinfusion for SAA treatment. To determine the treatment efficacy of infusing a certain subgroup of NK cells in combination with IST, in this study, we developed an immune attack‐mediated AA mouse model by total body irradiation (TBI) and allolymphocyte infusion. We have discussed the efficacy of TIM‐3(+) NK or TIM‐3(−) NK cell infusion in combination with IST in BMF/AA mice, which may further improve the treatment efficacy of patients with AA.

## 2 MATERIALS AND METHODS

### 2.1 Construction of BMF mouse model

Specific‐pathogen‐free grade and 8 weeks old CB6F1 mice (strain code: 303), except for those in the normal control (NC) group, were exposed to a sublethal TBI dose of 5Gy before lymph node cell infusion. Lymph node cells were infused from the groin, brachial plexus, and axilla of C57BL/6 mice. CB6F1 mice were injected with lymphocytes (5 × 10^7^ cells) through the caudal vein to induce BMF. The successfully constructed AA mouse model survived for more than 17 days. On the 17th day, mice were euthanized and blood samples were collected for further experiments.

### 2.2 Antibodies for flow cytometry (FCM)

The conjugated antibodies used for the detection of functional molecules are as follows: PE‐Vio770 mouse anti‐human CD56, PerCP‐Vio770 rat anti‐mouse T‐cell receptor, PE‐Vio770 rat anti‐mouse NK1.1, allophycocyanin (APC) rat anti‐mouse TIM‐3, PE rat anti‐mouse NK group (NKG)2A, and PE rat anti‐mouse NKG2D antibody, along with the isotype controls these were purchased from Miltenyi Biotec (Cologne, Germany). Mouse lysing solution was purchased from BD Pharmingen (Franklin Lakes, NJ, USA).

### 2.3 Isolation and purification of TIM‐3(+) and TIM‐3(−) NK cells

We collected the anticoagulated peripheral blood (with EDTA) of mice by eyeball extraction. Isolation of peripheral NK cells was performed using an NK cell isolation kit II mouse (Miltenyi Biotec, Germany). Peripheral TIM‐3(+) NK cells were isolated using TIM‐3 antiAPC microbeads (Miltenyi Biotec, Germany). The purity of TIM‐3(+) or TIM‐3(−) NK cells was over 90%, as detected by the multiparameter FCM (BD Biosciences), and analyzed using the Cell Quest software program (Version 3.1, Becton Dickinson).

### 2.4 Western blotting

Isolated NK, TIM‐3(+) NK, and TIM‐3(−) NK cells were collected and lysed directly in the radioimmunoprecipitation assay buffer supplemented with a complete protease inhibitor (Roche, Basel, Switzerland) and phosphatase inhibitors (Solarbio Science & Technology, Beijing, China). Protein levels in the lysates were quantified using a bicinchoninic acid assay kit. Proteins were separated in 4%–20% precast gels and transferred to nitrocellulose membranes (Pall Corporation, New York, NY, USA). The membranes were blocked with 10% skimmed milk (Chuntest Biotechnology, Shanghai, China) and subsequently incubated with anti‐phosphatidylinositol 3‐kinase (PI3K) p110α, anti‐protein kinase B (Akt), anti‐Phospho‐Akt (P‐Akt) and anti‐glyceraldehyde 3‐phosphate dehydrogenase (GAPDH) antibodies (Cell Signaling Technology, Danvers, MA, USA) at a dilution of 1:1000. The antibodies were dissolved in a solution containing 5% dried milk in Tris‐buffered saline with Tween 20 (20 mmol/L Tris–HCl buffer, pH 7.4, 150 mmol/L NaCl, and 0.05% Tween 20). After extensive washing with phosphate‐buffered saline, the membranes were then incubated with relevant horseradish peroxidase‐conjugated secondary antibodies (1:5000 dilution; Cell Signaling Technology). The labeled protein bands were detected using Super ECL Plus detection reagent. All protein levels were normalized to GAPDH.

### 2.5 Administration of immunosuppressive agents and/or NK cells transfusion to BMF mice

After AA induction, AA mice were divided into the following seven groups based on the given treatment: AA group, CsA (Zhongmei Huadong Pharmaceutical Co. LTD, Hangzhou, China) treatment group, TIM‐3(+) NK cells treatment group, TIM‐3(−) NK cells treatment group, CsA combined with TIM‐3(−) NK cells treatment (CsA + TIM3–NK) group, TIM‐3 blocker treatment (TIM‐3 blocker) group, and CsA combined with TIM‐3 blocker treatment (CsA + TIM‐3 blocker) group. Additionally, two control groups were formed, namely the NC and TBI groups. After infusion with donor lymphocytes for 17 days, the AA group was administered CsA (50 mg/kg/d, *n* = 10) or TIM‐3 blocker (eBioscience, San Diego, CA, USA; 20 μg, twice a week for 2 weeks). On the 1st and 8th day after lymphocyte infusion, some CB6F1 mice received allogeneic 10^7^ TIM‐3(+) or TIM‐3(−) NK cells transfusion to establish the BMF + NK mouse model. All mice were fed a healthy diet and then sacrificed on the 17th day. After treatment, the general condition, whole blood cell and BMC count, and immune condition of mice in each group were compared.

### 2.6 Weight, whole blood cell and BMC count and bone marrow biopsy histology in mice

Blood was collected from the retro‐orbital sinus, and BMCs were obtained by femoral cavity flushing. A whole blood cell count was performed using an automatic blood cell analyzer (MEK‐722, Nihon Kohden). We fixed the mice femurs with 4% paraformaldehyde overnight and sectioned them for paraffin embedding. Following this, hematoxylin–eosin staining was performed, and slides were observed under a light microscope (Nikon Diaphot, Tokyo, Japan) with a 10× objective lens.

### 2.7 The immune condition of mice

Blood was collected from the retro‐orbital sinus, and BMCs were obtained by femoral cavity flushing. Many circulating CD4^+^T and CD8^+^T cell subsets in mice were identified by FCM.

### 2.8 Statistical analyses

Data are presented as mean ± SD from at least three independent experiments. The significance between means was determined with multiple *t*‐tests using the HolmSidak method, one‐way analysis of variance (ANOVA) when Gaussian distribution was assumed, and Kruskal–Wallis test when Gaussian distribution was not assumed. Multiple pairwise comparison tests were performed between the control and BMF groups. Statistical analyses were performed using GraphPad Prism version 6.0 software and Statistical Package for the Social Sciences 21.0 statistical software. The *p*‐values of <.05 were considered statistically significant.

## 3 RESULTS

### 3.1 TIM‐3 expression in NK cells of AA mice was significantly lower than that of the TBI and NC group mice

**FIGURE 1:**
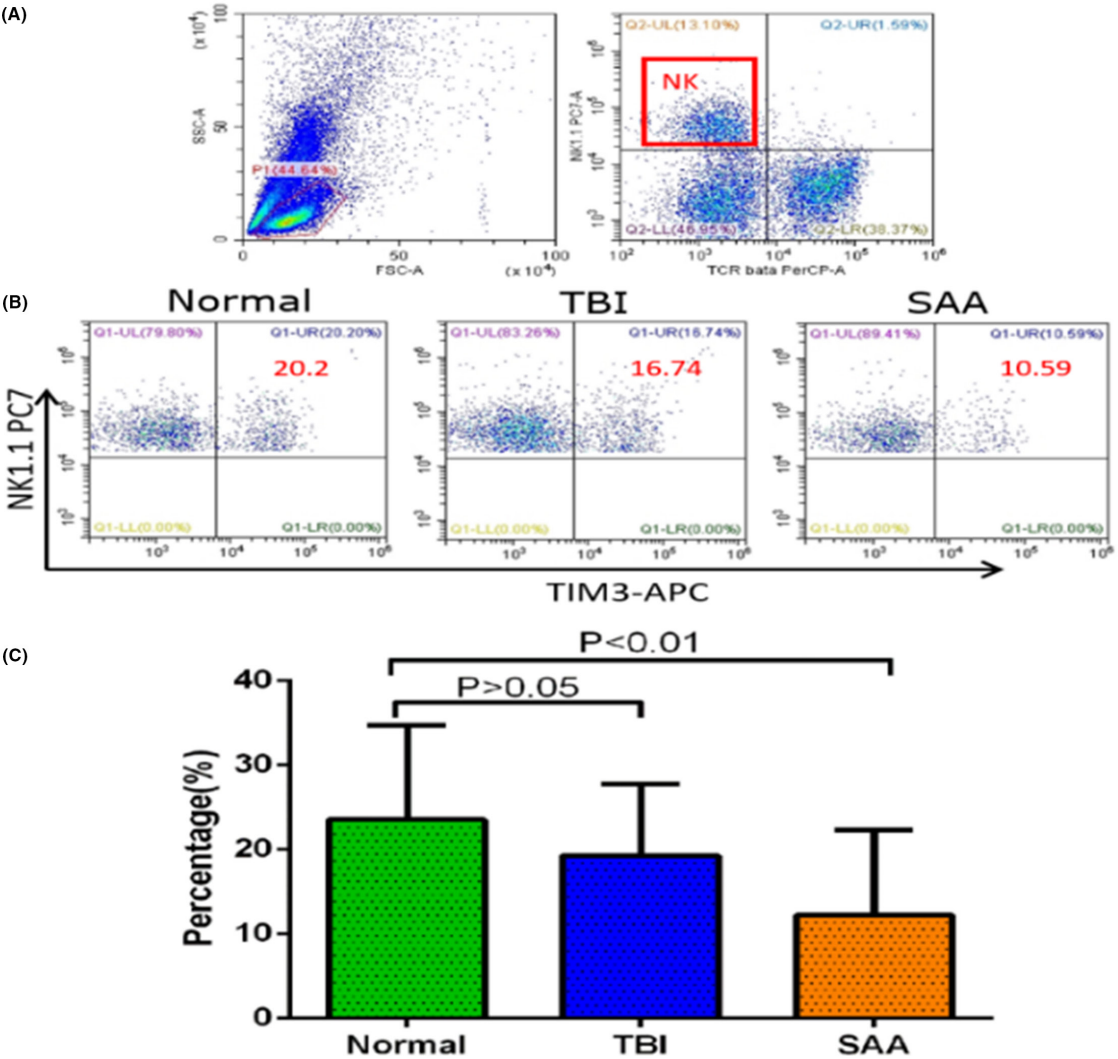
Comparison of TIM‐3 expression in peripheral blood NK cells of SAA, TBI, and normal mice is displayed as follows: (A) Flow cytometry detection of TCR^−^NK1.1^+^ gated mouse NK cells; (B) Comparison of TIM‐3 expression of NK cells in SAA mice, TBI mice, and normal mice; (C) Lower expression of TIM‐3 in NK cells of SAA mice than that of TBI mice (*p* < .05) and normal mice (*p* < .01).

TIM‐3 expression in the peripheral NK cells of AA mice was 12.21 ± 10.06%, which was significantly lower than that of the TBI (19.24 ± 8.52%, *p* < .01) and NC group mice (23.52 ± 11.17%, *p* < .01) (Figure 1). There was no statistically significant difference in TIM‐3 expression in NK cells between TBI and NC group mice (*p* > .05). Compared with the NC group, TIM‐3 expression in NK cells in AA mice was similar to those in patients with AA, as in both cases, TIM‐3 expression was lower than that of the NC group mice.

### 3.2 TIM‐3(−) NK cells in AA mice highly expressed activating receptors than TIM‐3(+) NK cells

**FIGURE 2:**
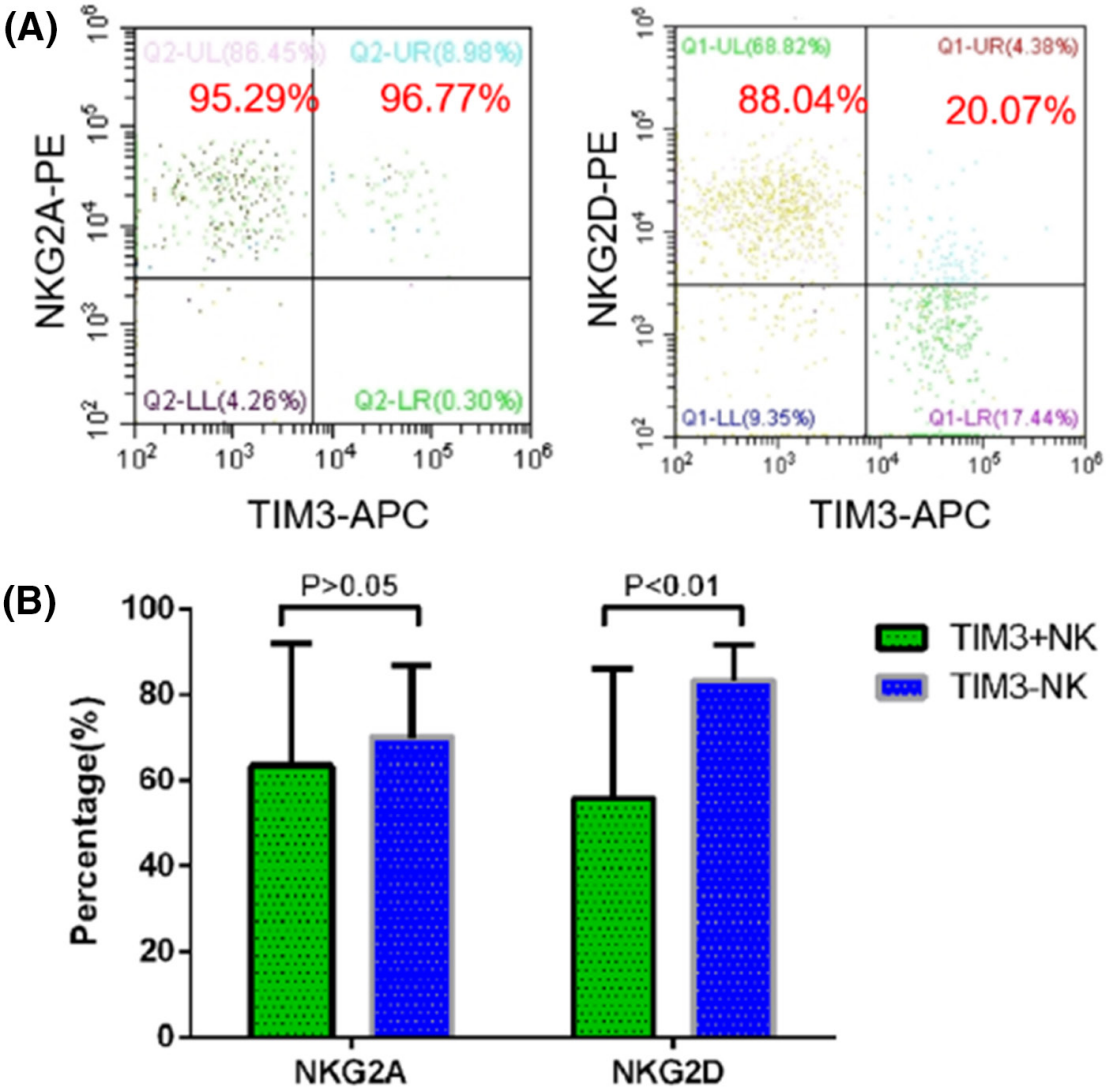
Comparison of expression of TIM‐3(+) NK and TIM‐3(−) NK cells in AA mice with NKG2A and NKG2Dis shown as follows: (A) Comparison of AA mice TIM‐3(+) NK and TIM‐3(−) NK cells against NKG2A and NKG2D expression by flow cytometry; (B) statistically indifferent expression of TIM‐3(−)NK cells inhibitory receptor NKG2A in SAA mice from that of TIM‐3(+) NK cells (*p* > .05); significantly higher expression of TIM‐3(−) NK cell activating receptor NKG2D than that of TIM‐3(+) NK cells (*p* < .01).

The expression of functional molecule inhibitory receptor NKG2A on TIM‐3(+) and TIM‐3(−) NK cells in AA mice was 63.44 ± 28.56% and 70.15 ± 16.89%, respectively (*p* > .05). The expression of activating receptor NKG2D on TIM‐3(+) and TIM‐3(−) NK cells in AA mice was 55.87 ± 40.24% and 83.38 ± 8.41%, respectively, with significantly higher expression in TIM‐3(−) NK cells (*p* < .05). These results suggested that the biological activity of TIM‐3(−) NK cells was stronger compared with that of TIM‐3(+) NK cells, and the changes in trends were similar to those of patients with AA (Figure 2).

### 3.3 The expression of PI3K, P‐Akt and P‐Akt/Akt ratio in TIM‐3(−) NK cells of AA mice was significantly higher than those in TIM‐3(+) NK cells

**FIGURE 3:**
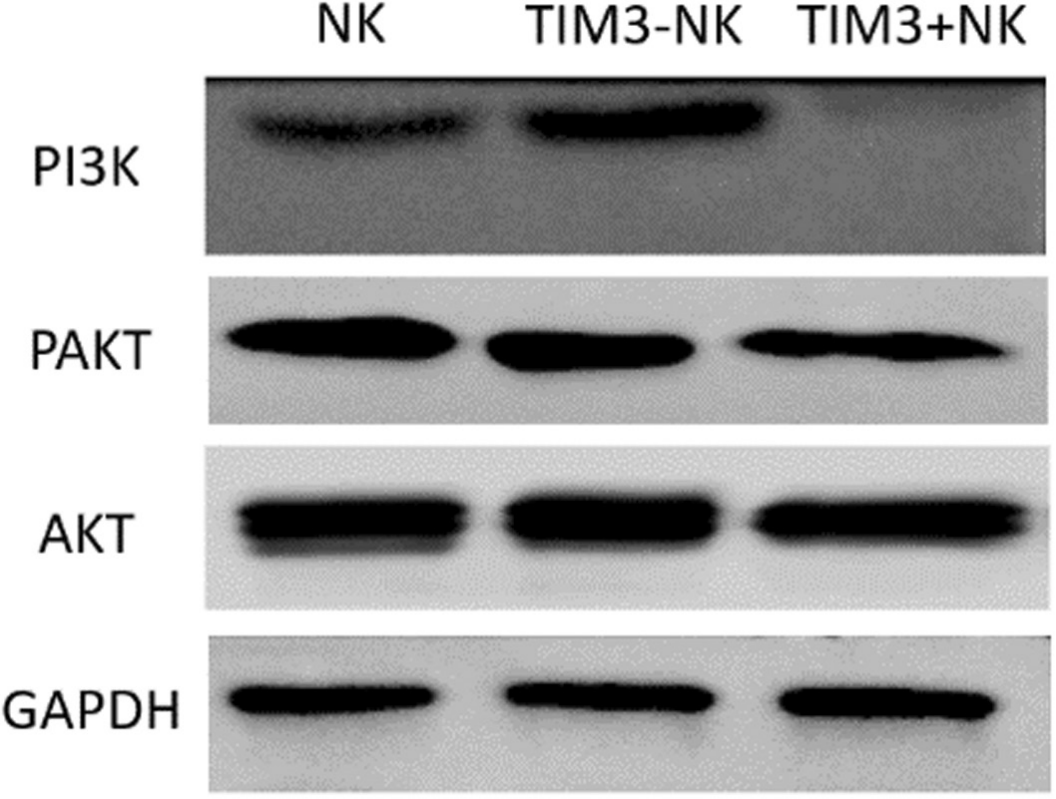
Comparison of expression of pathway proteins in aplastic anemia (AA) mice TIM‐3(−) NK and TIM‐3^+^NK cell receptor: AA mouse model NK cells, TIM‐3^−^NK, and TIM‐3^+^NK cell receptor signal pathway protein AKT levels are not significantly different compared to P‐AKT in NK cells. TIM‐3(−) NK cells had no significant difference but were significantly higher than TIM‐3(+) NK cells, and the expression of PI3K in TIM‐3(−) NK, NK, and TIM‐3(+) NK cells gradually decreased. The biological activity of TIM‐3(−) NK cells was higher than that of TIM‐3(+) NK cells.

The analyses of the relative gray value of the samples showed that the relative expression of PI3K in NK, TIM‐3(−) NK and TIM‐3(+) NK cells in AA mice were 0.59 ± 0.12%, 1.33 ± 0.11% and 0.07 ± 0.04%; the relative expression of Akt was 1.61 ± 0.14%, 1.65 ± 0.14% and 1.49 ± 0.16%; and the relative expression of P‐Akt was 1.44 ± 0.14%, 1.32 ± 0.13% and 0.89 ± 0.15%, respectively. Akt levels in TIM‐3(−) NK cells of AA mice were similar to that in TIM‐3(+) NK cells (*p* > .05); however, PI3K and P‐Akt levels and P‐Akt/Akt ratio in TIM‐3(−) NK cells were significantly higher than those in TIM‐3(+) NK cells (*p* < .05). Although the total NK cells partially expressed TIM‐3, they were not significantly affected by post‐in vivo signaling pathway proteins, such as Akt and P‐Akt, when compared with TIM‐3(−) NK cells (*p* > .05). Compared with total NK cells, PI3K expression was lower than that in TIM‐3(−) NK cells (*p* < .05) but higher than that in TIM‐3(+) NK cells (*p* < .05). The results suggested comparatively higher biological activity of TIM‐3(−) NK cells compared with TIM‐3(+) NK cells and the inhibitory role of TIM‐3 against NK cell activity (Figure 3).

### 3.4 The general condition of AA mice improved significantly after TIM‐3(−) NK cell infusion and CsA + TIM‐3(−) NK cell infusion treatment

**FIGURE 4:**
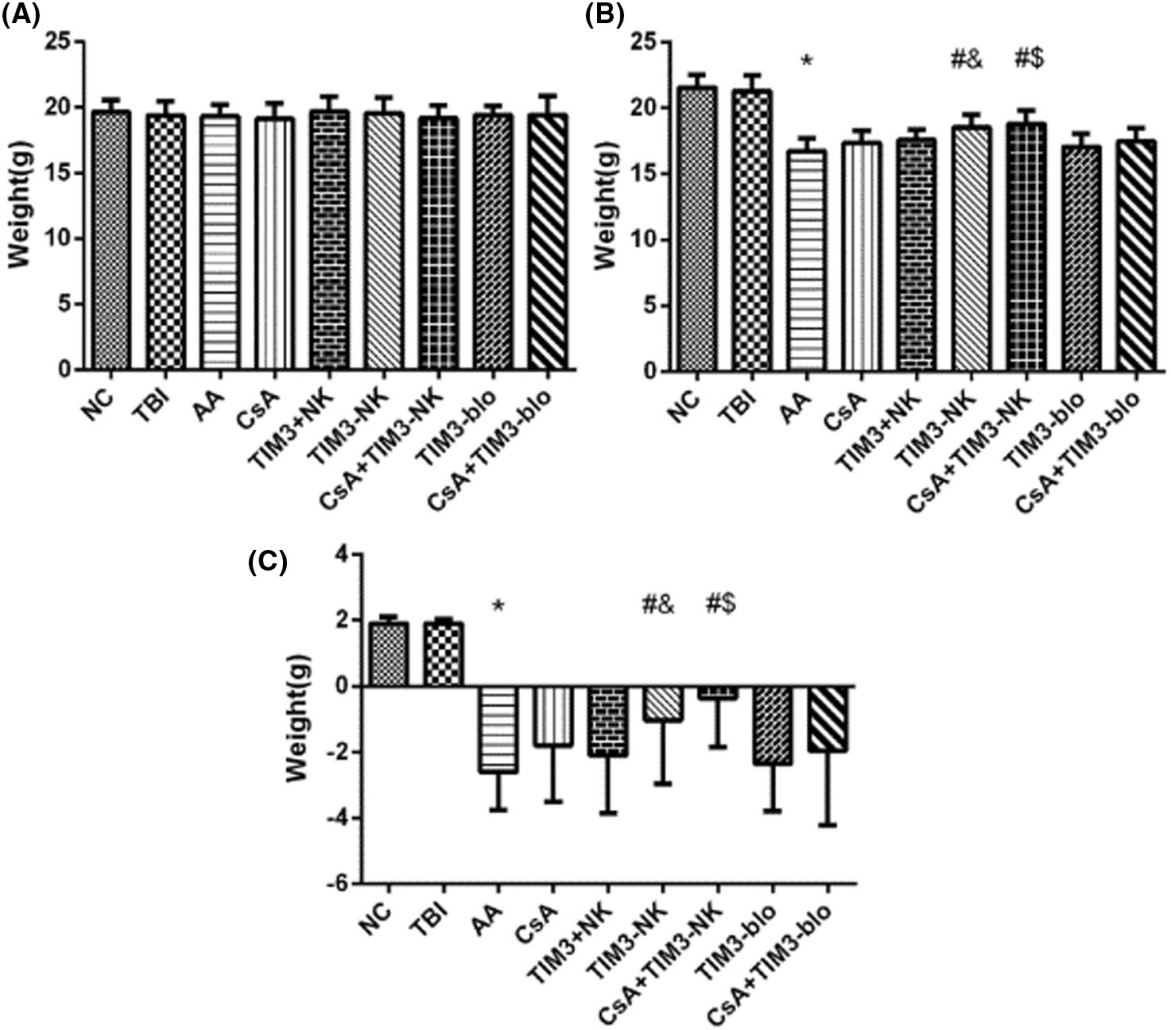
Body weight of mice in each group before and after modeling is displayed as follows: (A) body weight of mice in each group before modeling, (B) body weight of mice in each group after modeling, and (C) difference in body weight between 17 days after modeling and before modeling. *Compare with NC and TBI group (*p* < .05). ^#^Compare with aplastic anemia (AA) group (*p* < .05). ^$^Compare with CsA treatment group (*p* < .05).

There was no body weight difference before TBI and lymphocyte infusion among all groups (Table 1, Figure 4A, and all *p* > .05). The NC and TBI group mice gained slight weight on the 17th day, whereas the weight of mice in the AA group was significantly lower than the basal weight on the 17th day compared with the NC group (*p* < .01).

**TABLE 1:** Analysis of the weight of mice in each group on the 17th day (X ± SD).

	*n*	Weight on the first day after modeling(g)	Weight on the 17th day after modeling(g)	△d17‐d1 Median(g)
NC	10	19.65 ± 0.92	21.55 ± 0.95	1.9
TBI	10	19.39 ± 1.09	21.29 ± 1.17	1.9
AA	10	19.32 ± 0.88	16.72 ± 0.98^a^	−2.7^a^
CsA	10	19.14 ± 1.17	17.35 ± 0.90	−1.35
TIM3 + NK	10	19.67 ± 1.16	17.57 ± 0.80	−1.75
TIM3‐NK	10	19.56 ± 1.20	18.53 ± 0.98^b^	−1.15^b^
CsA+ TIM3‐NK	10	19.17 ± 1.01	18.81 ± 0.99^b^ ^,^ ^c^	−0.2^b^ ^,^ ^c^
TIM3 blocker	6	19.40 ± 0.72	17.05 ± 0.99	−2.2
CsA + TIM3 blocker	6	19.42 ± 1.46	17.47 ± 1.02	−1.18

Abbreviations: AA, aplastic anemia; CsA + TIM‐3 blocker, CsA combined with TIM‐3 blocker treatment; CsA + TIM‐3(−) NK, CsA combined with TIM‐3(−) NK cell treatment; CsA, CsA treatment; NC, Normal control; TBI, total body irradiation; TIM‐3 blocker, TIM‐3 blocker treatment; TIM‐3(−) NK, TIM‐3(−) NK cell treatment; TIM‐3(+) NK, TIM‐3(+) NK cell treatment.

^a^ Compare with NC and TBI group (*p* < .05).

^b^ Compare with AA group (*p* < .05).

^c^ Compare with CsA treatment group (*p* < .05).

On the 17th day, compared with the AA group, CsA treatment group, TIM‐3(+) and TIM‐3(−) NK cell treatment group, CsA + TIM‐3(−) NK group, and CsA + TIM‐3 blocker group had less body weight loss (all *p* < .05 and Figure 4B). However, the weight did not return to the NC group level, and there was no significant change in body weight gain in the TIM‐3 blocker group. Compared with the AA group, the weight gain in the TIM‐3(−) NK cell treatment group and the CsA + TIM‐3(−) NK group was the most significant (all *p* < .05). Compared with the TIM‐3(+) NK cell treatment group, the body weight in TIM‐3(−) NK cell treatment group had no significant weight gain (*p* > .05). Compared with the CsA treatment group, the weight of mice in the CsA + TIM‐3(−) NK group was higher (*p* < .05). The weight of mice in the CsA + TIM‐3 blocker group had no significant weight change compared with the CsA treatment group (*p* > .05, and Figure 4C).

The results showed that the general state of BMF mice was significantly worse compared with that of NC and TBI mice. Regardless of the fur, the mobility of the BMF mice was worse compared with that of NC mice. Although the body weight increased after treatment, it could not return to normal levels in the short term. Among all, TIM‐3(−) NK cell treatment group and CsA + TIM‐3(−) NK group had the best effect.

### 3.5 The whole blood cell count of AA mice improved significantly after TIM‐3(−) NK cell infusion and CsA + TIM‐3(−) NK cell infusion treatment

**FIGURE 5:**
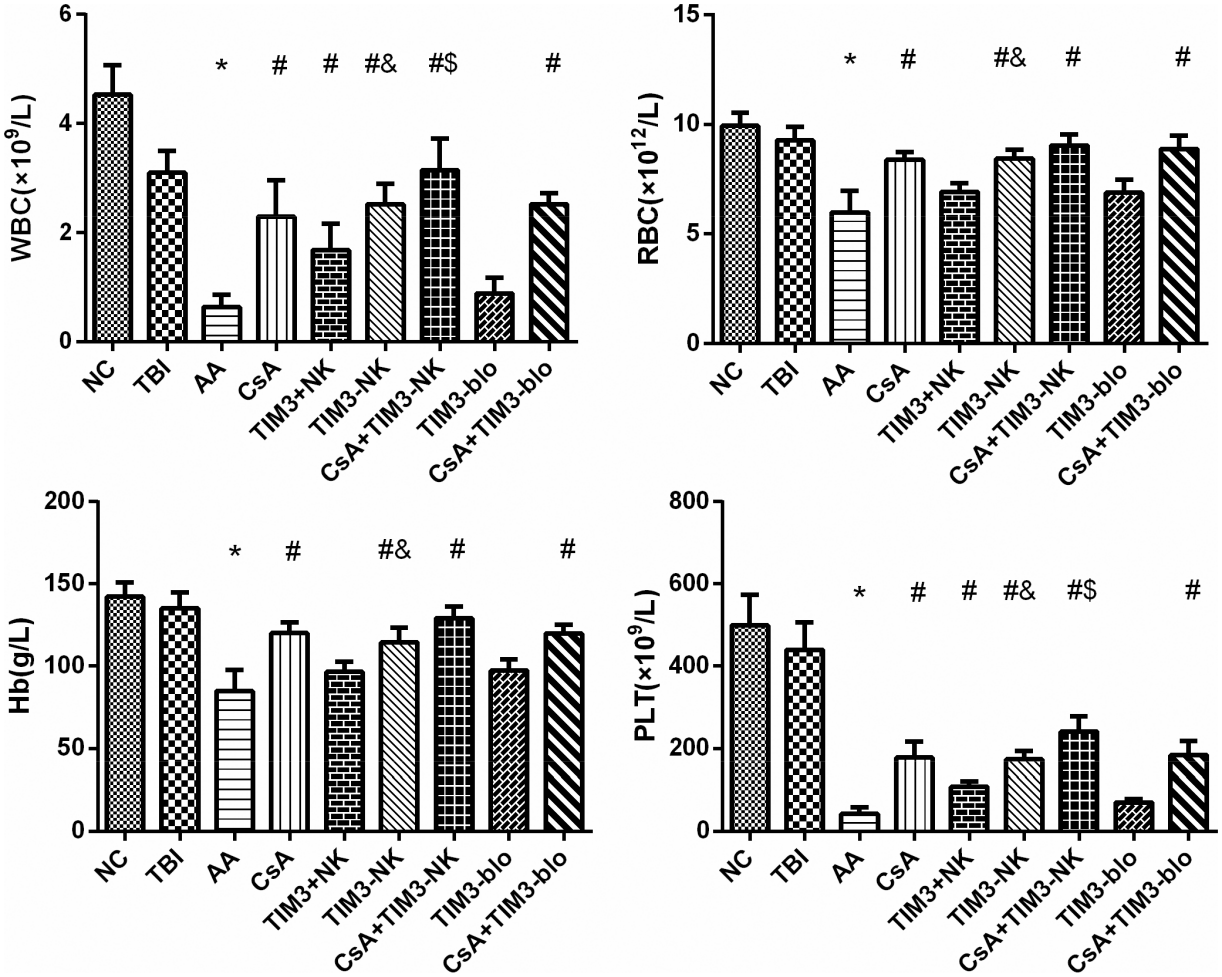
Blood counts of mice on the 17th day after modeling; *Compare with NC and TBI group (*p* < .05). ^#^Compare with AA group (*p* < .05). ^$^Compare with CsA treatment group (*p* < .05). ^&^Compare with TIM‐3(+) NK cell treatment group (*p* < .05).

The whole blood cell count on the 17th day after modeling is shown in Table 2. Pairwise comparison showed that compared with the NC group, the whole blood cell count in the AA group was significantly decreased (*p* < .01). Compared with the AA group, the whole blood cell count of the CsA treatment group, TIM‐3(−) NK cell treatment group, CsA + TIM‐3(−) NK group, and CsA + TIM‐3 blocker group significantly increased (*p* < .05). Compared with the AA group, only white blood cell (WBC) and platelet (PLT) counts were statistically different in TIM‐3(+) NK cell treatment group (*p* < .05). WBC, red blood cell (RBC), hemoglobin (Hb), and PLT of the TIM‐3 blocker group showed a statistically insignificant increasing trend (*p* > .05). The improvement of the hemogram of the TIM‐3(−) NK cell treatment group was significantly better than that of the TIM‐3(+) NK cell treatment group (*p* < .05). WBC, RBC, Hb, and PLT increased in the CsA + TIM‐3(−) NK group compared with the CsA treatment group, and the WBC and PLT significantly increased with a statistically significant difference (*p* < .05). The whole blood cell count of the CsA + TIM‐3 blocker group slightly increased compared with the CsA treatment group without any statistical difference (*p* > .05) (Figure 5). The results suggested that the whole blood cell count of AA mice recovered after NK cell reinfusion, especially in the TIM‐3(−) NK cell treatment group. Furthermore, there was a certain synergistic effect with the combined treatment of CsA. Further studies on dose and course adjustment are needed to optimize the effect. However, after the treatment, the blood cell counts of the mice in each group were still lower than those in the NC group, indicating that it would take longer for the mice to return to normal whole blood cell count.

**TABLE 2:** Analysis of blood cell count of mice in each group on the 17th day (X ± SD).

	*n*	WBC (×10^9^/L)	RBC (×10^12^/L)	Hb (g/L)	PLT (×10^9^/L)
NC	10	4.53 ± 0.54	9.93 ± 0.60	142.30 ± 8.81	499.8 ± 73.62
TBI	10	3.1 ± 0.40	9.29 ± 0.61	135 ± 9.98	439.8 ± 67.26
AA	10	0.64 ± 0.22^a^	5.99 ± 0.98^a^	84.9 ± 12.87^a^	42.3 ± 16.71^a^
CsA	10	2.3 ± 0.66^b^	8.38 ± 0.37^b^	120.1 ± 6.49^b^	177.7 ± 38.81^b^
TIM‐3(+)NK	10	1.68 ± 0.48^b^	6.92 ± 0.41	96.50 ± 6.26	108.4 ± 12.38^b^
TIM‐3(−) NK	10	2.52 ± 0.38^b^ ^,^ ^d^	8.48 ± 0.4^b^ ^,^ ^d^	114.6 ± 8.78^b^ ^,^ ^d^	173.5 ± 21.14^b^ ^,^ ^d^
CsA + TIM‐3(−) NK	10	3.14 ± 0.58^b^ ^,^ ^c^	9.04 ± 0.51^b^ ^,^ ^c^	129.2 ± 7.13^b^ ^,^ ^c^	241.1 ± 36.65^b^ ^,^ ^c^
TIM‐3 blocker	6	0.88 ± 0.29	6.88 ± 0.58	97.33 ± 6.83	70.67 ± 7.45
CsA + TIM‐3 blocker	6	2.52 ± 0.20^b^	8.89 ± 0.59^b^	119.8 ± 5.53^b^	183.7 ± 34.51^b^

Abbreviations: AA, aplastic anemia; CsA + TIM‐3 blocker, CsA combined with TIM3 blocker treatment; CsA + TIM‐3(−) NK, CsA combined with TIM‐3(−) NK cell treatment; CsA, CsA treatment; NC, Normal control; TBI, total body irradiation; TIM‐3 blocker, TIM3‐ blocker treatment; TIM‐3(−) NK, TIM‐3‐NK cell treatment; TIM‐3(+) NK, TIM‐3(+) NK cell treatment.

^a^ Compare NC and TBI group (*p* < .05).

^b^ Compare with AA group (*p* < .05).

^c^ Compare with CsA treatment group (*p* < .05).

^d^ Compare with TIM‐3(+) NK cell treatment group (*p* < .05).

### 3.6 BMC count and histopathology of AA mice improved significantly after TIM‐3(−) NK cell infusion and CsA + TIM‐3(−) NK cell infusion treatment

**FIGURE 6:**
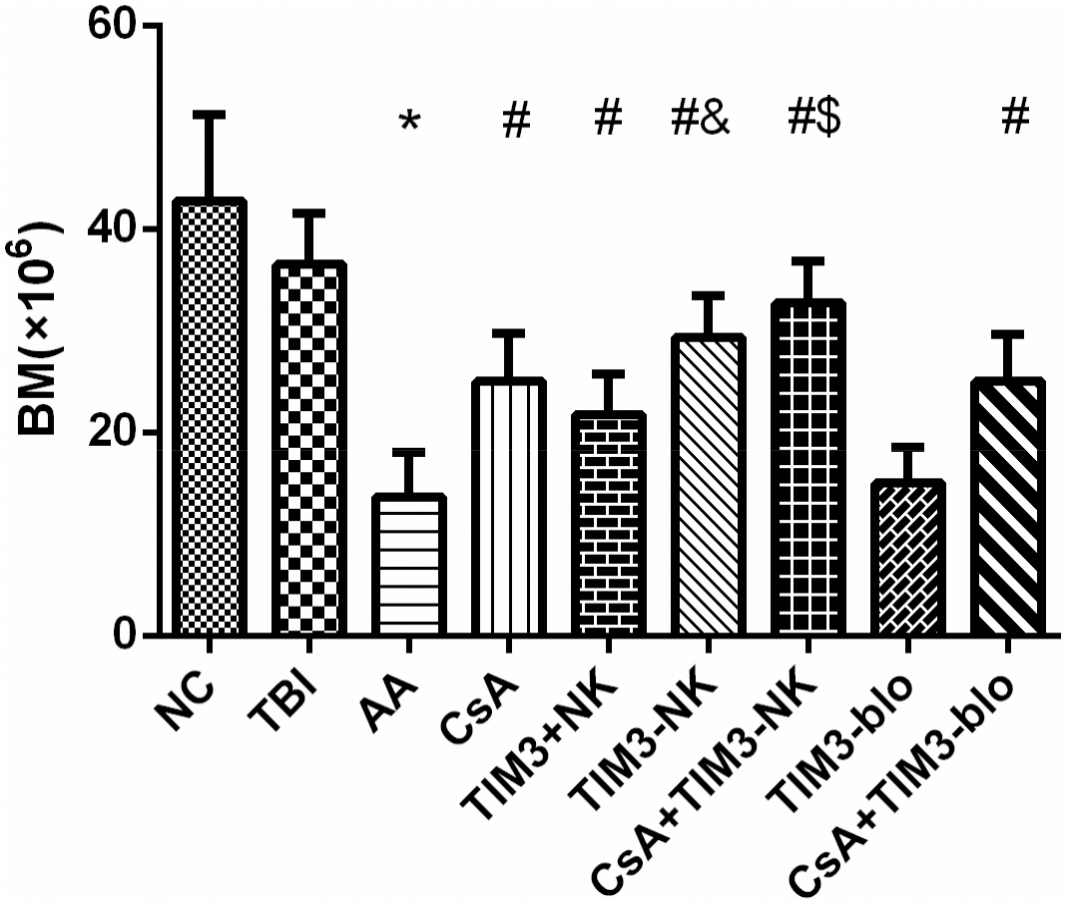
Bone Marrow counts of mice on the 17th day after modeling. *Compare with NC group (*p* < .05). ^#^Compare with aplastic anemia (AA) group (*p* < .05). ^$^Compare with CsA treatment group (*p* < .05). ^&^Compare with TIM‐3(+) NK cell treatment group (*p* < .05).

BMC count in the AA group was significantly lower than that in the NC group, indicating that the modeling was successful. A pairwise comparative ANOVA showed that the BMC count of the CsA treatment group, TIM‐3(+) NK and TIM‐3(−) NK cell treatment groups, the CsA + TIM‐3(−) NK group, and CsA + TIM‐3 blocker group were all increased compared with the AA group (*p* < .05). BMC count in the TIM‐3 blocker group was not significantly higher than that of the AA group (*p* > .05), suggesting that the CsA treatment group, TIM‐3(+) and TIM‐3(−) NK cell treatment groups, CsA + TIM‐3(−) NK group, and CsA + TIM‐3 blocker group treatments were effective, whereas the treatment of the TIM‐3 blocker group was not effective. Compared with the TIM‐3(+) NK cell treatment group, the BMC count of the TIM‐3(−) NK cell treatment group was significantly higher (*p* < .05), suggesting that the TIM‐3(−) NK cell treatment was better than the TIM‐3(+) NK cell treatment and had better efficacy. BMC count of the CsA + TIM‐3 blocker group had no significant increase in cell count compared with that of the CsA treatment group (*p* > .05). The CsA treatment group, TIM‐3(+) and TIM‐3(−) NK cell treatment groups, CsA + TIM‐3(−) NK group, and CsA + TIM‐3 blocker group had higher BMC counts than the AA group but were still lower than that of the NC group (*p* < .05). Among the TBI, TIM‐3(−) NK cell treatment and CsA + TIM‐3(−) NK groups, the remaining two groups had a lower BMC count than the TBI group without any statistically significant difference (*p* > .05 and Figure 6). The results above suggest that BMC count in the mice did not recover to the normal level after treatment in each group, and it takes time to recover. The TIM‐3(−) NK cell treatment group and CsA + TIM‐3(−) NK group had better effects, especially the CsA + TIM‐3(−) NK group.

The right femur of mice from each group was taken, and the effects of CsA, CsA + TIM‐3(−), TIM‐3(+) NK cell infusion therapy, and TIM‐3 blocker treatment on the proportion of hematopoietic tissues in mice were compared by bone marrow histopathology.

**FIGURE 7:**
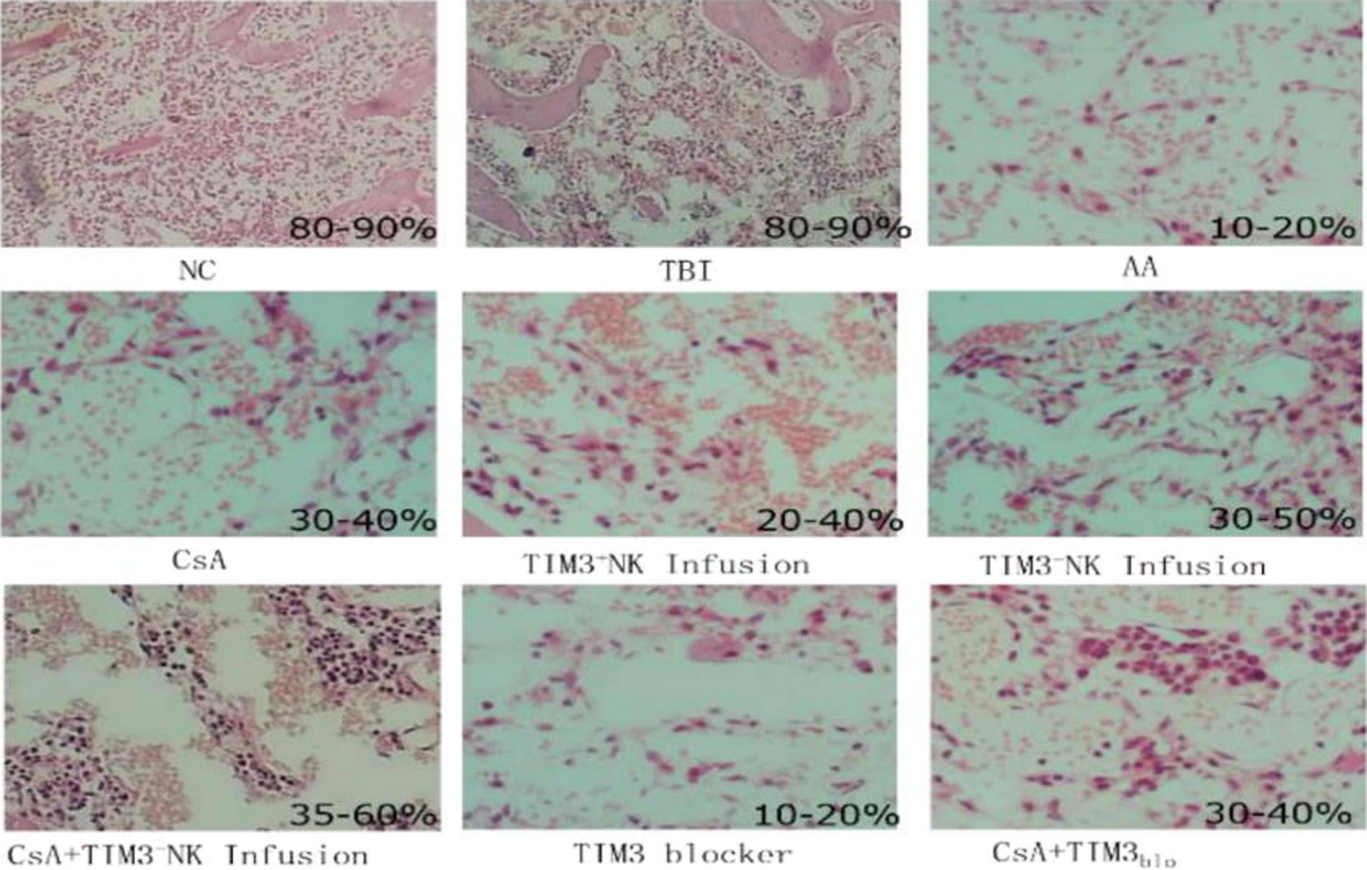
Bone marrow histopathology in each group (HE staining, 10×): the BM histopathology of aplastic anemia mice improved significantly after TIM‐3(−) NK cell infusion and CsA combined with TIM‐3(−) NK cell infusion treatment.

The results showed that the bone marrow hematopoietic area of AA mice was significantly lower than that of NC mice, whereas CsA treatment and TIM‐3(+) and TIM‐3(−) NK cell infusion therapy increased the bone marrow hematopoietic area of AA mice. TIM‐3(−) NK cell infusion therapy was better than the TIM‐3(+) NK cell infusion therapy, as the bone marrow hematopoietic area of AA mice increased significantly in the CsA + TIM‐3(−) NK group (Figure 7).

### 3.7 The immune condition of AA mice recovered after the treatment of TIM‐3(−) NK cell reinfusion

**FIGURE 8:**
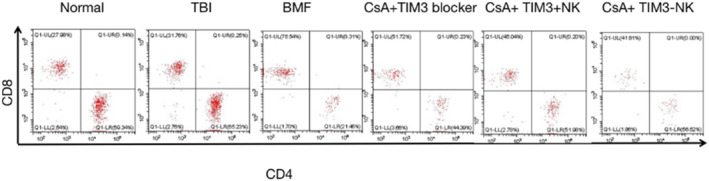
Comparison of immune cells in each group expression by flow cytometry: the immune condition of aplastic anemia (AA) mice recovered after the treatment of TIM‐3(−) NK cell reinfusion.

The CD4^+^T cell count, CD8^+^T cell count and CD4^+^T/CD8^+^T ratio on day 17 after modeling is shown in Table 3. Pairwise comparison showed that compared with the NC group, the CD4^+^T cell count and CD4^+^T/CD8^+^T ratio in the AA group was significantly decreased (*p* < .05), whereas the CD8^+^T cell count in the AA group was remarkably increased, with significant differences (*p* < .05). Compared with the AA group, the CsA treatment group, the CsA + TIM‐3 blocker group, CsA + TIM‐3(−) NK group, and CsA + TIM‐3(+) NK group all had significantly increased CD4^+^T cell count and decreased CD8^+^T cell count (*p* < .05). Compared with the CsA group, the CD4^+^T cell count and CD4^+^T/CD8^+^T ratio in CsA + TIM‐3(−) NK group was significantly higher than that in other groups (*p* < .05). The CD4^+^T and CD8^+^T cell count of the CsA + TIM‐3 blocker treatment group slightly changed compared with the CsA group but had no statistical difference (*p* > .05) (Figure 8). The CD8^+^T cell count in CsA + TIM‐3(−) NK group was lower than that in other groups (*p* < .05). The CD4^+^T cell count and CD4^+^T/CD8^+^T ratio increased in the CsA + TIM‐3(−) NK group compared with the CsA + TIM‐3 blocker group; however, the CD8^+^T cell count was decreased in the CsA + TIM‐3(−) NK group compared with the CsA + TIM‐3 blocker group. It was suggested that the immune condition of AA mice recovered after the treatment with TIM‐3(−) NK cell reinfusion. In addition, the combined treatment of CsA had a certain synergistic effect. However, the immune cell count of the mice in each group remained lower than that in the NC group after the treatment, indicating that it would take longer for the mice to return to normal levels of complete blood count (Figure 8).

**TABLE 3:** Analysis of immune cell count of mice in each group on the 17th day (X ± SD).

	*n*	CD4^+^T(%)	CD8^+^T(%)	CD4^+^T/CD8^+^T
NC	10	64.75 ± 2.23	31.46 ± 1.82	2.06 ± 0.12
TBI	10	62.21 ± 1.46	29.87 ± 1.83	2.09 ± 0.15
AA	10	29.47 ± 2.83^a^	68.03 ± 3.15^a^	0.43 ± 0.04^a^
CsA	10	44.05 ± 2.75^b^	50.78 ± 3.41^b^	0.87 ± 0.09^b^
CsA+ TIM3‐NK	10	50.46 ± 3.24^b^ ^,^ ^c^	41.77 ± 1.97^b^ ^,^ ^c^	1.21 ± 0.12^b^ ^,^ ^c^
CsA+ TIM3 + NK	6	45.31 ± 2.15	46.49 ± 1.48	0.98 ± 0.05
CsA + TIM3 blocker	6	42.50 ± 2.99^b^	51.59 ± 2.83^b^	0.83 ± 0.06^b^

Abbreviations: AA, aplastic anemia; CsA + TIM‐3 blocker, CsA combined with TIM‐3 blocker treatment; CsA + TIM‐3(−) NK, CsA combined with TIM‐3(−) NK cell treatment; CsA, CsA treatment; NC, Normal control; TBI, total body irradiation; TIM‐3 blocker, TIM‐3 blocker treatment; TIM‐3(−) NK, TIM‐3(−) NK cell treatment; TIM‐3(+) NK, TIM‐3(+) NK cell treatment.

^a^ Compare NC and TBI group (*p* < .05).

^b^ Compare with AA group (*p* < .05).

^c^ Compare with CsA treatment group (*p* < .05).

## 4 DISCUSSION

To further explore the immune pathogenesis of SAA and improve the therapeutic effect, the present study conducted mouse modeling experiments on the basis of previous studies.^9^, ^10^ An immune‐mediated AA mouse model was established by injecting MHC‐incompatible cytotoxic T lymphocytes into the mice to attack hematopoietic cells in the bone marrow and induce severe pancytopenia in mice. An increase in the number of CD8^+^T cells and the secretion of perforin and granular enzyme functional molecules were detected in this mouse model. At the same time, lymphocyte type I (such as IL‐2 and IFN‐*γ*.) was significantly increased, which was related to the internal environment of patients with SAA. There were many similarities between them, and the number and function of other immune indicators, such as NK cells, Treg cells and mDC subsets, were extensively similar.^11^, ^12^, ^13^ Some studies have reported that the ATG treatment of AA mice can effectively improve the blood images of the AA mouse model and restore its immune state, but special attention must be paid to the timing of ATG infusion therapy.^14^ Young et al.^15^ treated the AA mouse model with CsA and rapamycin and achieved remarkably positive results. These immunosuppressive drugs could not only improve the blood and bone marrow images of AA mice but also affect AA mice to different degrees. The mechanism of immune recovery provided an important reference value for the study of drug application. Presently, it has been discovered that blood and bone marrow functions of AA mice can be improved by inhibiting the super cellular immune function of AA mice or increasing the number of Treg cells to increase the immune tolerance of the mice.^16^, ^17^ Therefore, it is reasonable to study the pathogenesis of AA, the efficacy of the drug, and cell transfusion through this type of AA mouse model. In this study, we successfully constructed an immune‐mediated AA mouse model by irradiating the whole body of mice and injecting them with MHC‐incompatible lymphocytes.^18^, ^19^, ^20^, ^21^ The weight, peripheral blood, BM cell count, and immune condition of the AA mouse model were significantly lower than those of NC and TBI mice. Moreover, histopathology of the bone marrow indicated that the degree of hyperplasia of AA mice was severely decreased, red blood cells were rare, and megakaryocytes were not seen. It suggested that the mice have a hematopoietic failure, and the modeling is successful. Our previous research found that the number of NK cells in patients with SAA decreased, and the expression of activated receptors increased, which may play a protective role in the pathogenesis of SAA and inhibit the immune waterfall activation.^9^, ^10^ We speculated that the infusion of TIM‐3(−) NK cells might improve the efficacy of patients with SAA. In theory, TIM‐3 blockers could specifically block TIM‐3 expression on NK cells, decrease the proportion of NK cells, and treat patients with SAA.

The mice of the SAA model were divided into the NC group, TBI group, AA group, CsA treatment group, TIM‐3(+) NK cells treatment group, TIM‐3(−) NK cells treatment group, CsA + TIM‐3(−) NK group, TIM‐3 blocker treatment group, and CsA + TIM3 blocker treatment group. After treatment, the general condition, blood routine, bone marrow count, and pathological changes of mice in each group were compared. Our results reported that the CsA treatment group and the TIM‐3(−) NK cell treatment group could improve the weight of AA mice and promote the recovery of blood and bone marrow. However, the improvement of TIM‐3(+) NK cell treatment is relatively small. The therapeutic effect of TIM‐3(−) NK cell infusion is similar to that of CsA. Moreover, the therapeutic effect of CsA + TIM‐3(−) NK cell infusion is remarkable. It can significantly improve the general condition of AA mice and restore blood and bone marrow. Although the blood routine values were partially increased, the efficacy was unstable, which may be related to the expression of TIM‐3 as an NK cell‐non‐specific functional molecule in mice or related to the inhibitory effect of the blocker on other immune cells, resulting in the offset of the efficacy and side effects. Through TIM‐3(−) NK cell reinfusion treatment, we may improve the condition of AA mice and restore blood by possibly increasing the number of NK cells and suppressing the immune waterfall of AA. However, the mechanism of TIM‐3(−) NK cells regulating AA immunity and improving blood and bone marrow condition remains to be further explored. This study provides new treatment ideas for further improving the immune pathogenesis of patients with SAA and improving the efficacy of SAA treatment.

To conclude, through the study of animal models, TIM‐3(−) NK cell infusion therapy may have some synergy with CsA. Our findings allow the development of new therapeutic strategies based on TIM‐3(−) NK cell infusion treatment for SAA.

## AUTHOR CONTRIBUTIONS

All authors contributed to the study's conception and design. Material preparation, data collection and analysis were performed by Shaoxue Ding, Tian Zhang, Zixuan Liu, Yi Cui, Chunyan Liu and Rong Fu. The first draft of the manuscript was written by Shaoxue Ding and Zixuan Liu. All authors commented on previous versions of the manuscript. All authors read and approved the final manuscript.

## FUNDING INFORMATION

This study was supported by grants from the National Natural Science Foundation of China (8227010826), Tianjin Municipal Education Commission Scientific Research Project (2019KJ199), and Tianjin Science and Technology Plan Project (21JCZDJC01180).

## CONFLICT OF INTEREST STATEMENT

All authors report no conflicts of interest. The authors alone are responsible for the content and writing of this article.

## INFORMED CONSENT

Informed consent was obtained from all individual participants included in the study.

## Data Availability

Data that support the findings of this study are available from the corresponding author, Rong Fu, upon reasonable request.
